# Supplementary material for: Molecular diagnosis of scabies using a novel probe-based polymerase chain reaction assay targeting high-copy number repetitive sequences in the Sarcoptes scabiei genome
Source: PLoS Negl Trop Dis. 2021 Feb 24;15(2):e0009149. doi: 10.1371/journal.pntd.0009149 (PMC7939366; doi:10.1371/journal.pntd.0009149)
Supplement: S1 Fig — qPCR amplification curves of (A) SSR5 and (B) SSR6 generated from serial 10-fold linearised plasmid DNA template dilutions ranging from 3 × 10^8^ to 3× 101 copies/μL. Each dilution was tested in triplicate. Blue line across indicates positive threshold. Linear standard curves of (C) the SSR5 assay and (D) the SSR6 assay constructed with triplicate cycle quantification (Cq) values against log starting DNA template quantity of each dilution. The equation for the regression line and the coefficient of determination (R2) value are shown in the graphs. Detection limit was found to be at 3.8 copies per 10 μL of reaction for the SSR5 assay and 1.9 copies per 10 μL of reaction for the SSR6 assay. *RFU: relative fluorescence unit. (PDF) [file pntd.0009149.s001.pdf]

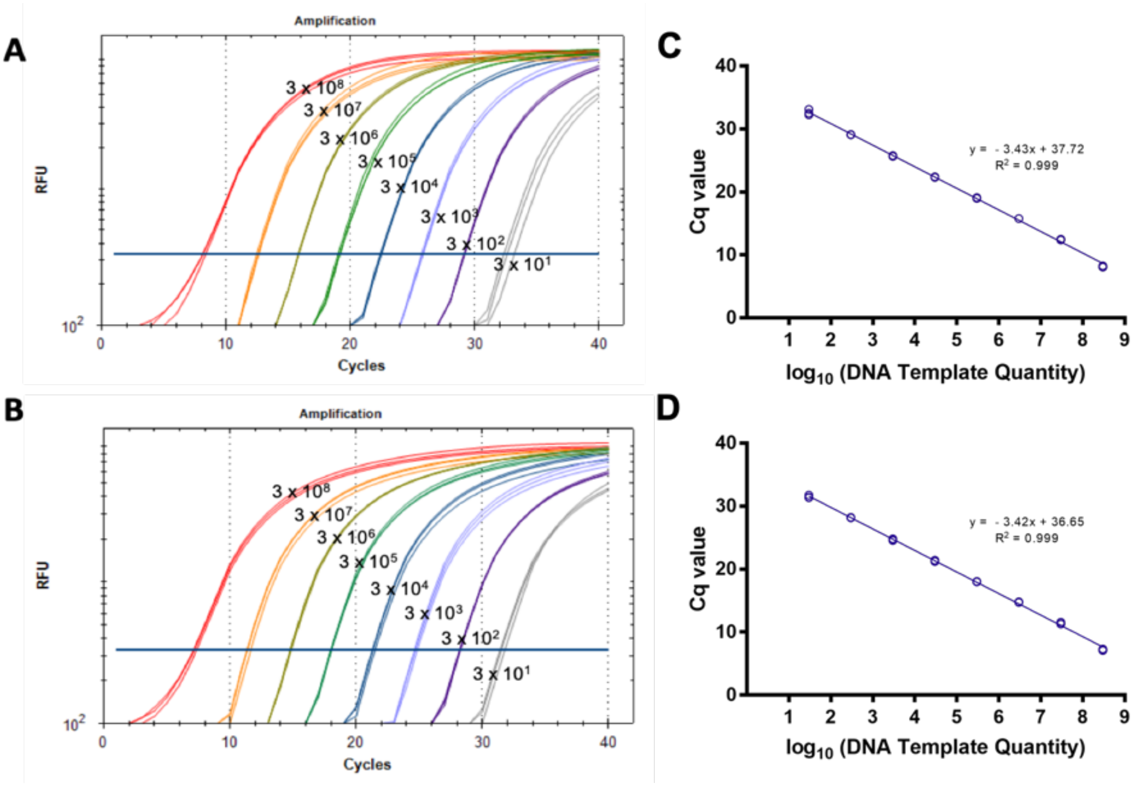

S1 Fig. Probe-based qPCR assays detection limits and efficiency. qPCR amplification curves of **(A)** SSR5 and **(B)** SSR6 generated from serial 10-fold linearised plasmid DNA template dilutions ranging from 3×10^8 to 3×10^1 copies/µL. Each dilution was tested in triplicate. Blue line across indicates positive threshold. Linear standard curves of **(C)** the SSR5 assay and **(D)** the SSR6 assay constructed with triplicate cycle quantification (Cq) values against log starting DNA template quantity of each dilution. The equation for the regression line and the coefficient of determination (R^2) value are shown in the graphs. Detection limit was found to be at 3.8 copies per 10 µL of reaction for the SSR5 assay and 1.9 copies per 10 µL of reaction for the SSR6 assay. *RFU: relative fluorescence unit
