# Supplementary material for: Molecular diagnosis of scabies using a novel probe-based polymerase chain reaction assay targeting high-copy number repetitive sequences in the Sarcoptes scabiei genome
Source: PLoS Negl Trop Dis. 2021 Feb 24;15(2):e0009149. doi: 10.1371/journal.pntd.0009149 (PMC7939366; doi:10.1371/journal.pntd.0009149)
Supplement: S2 Fig — Amplification (A) SSR5 and (B) SSR6 with gDNA panel consisting of human skin, pig skin, P. humanus, D. canis, P. ovis and serially-diluted HDMs. Neat, 1:10, 1:100 dilutions replicate presence of 100 (HDM100), ten (HDM10) and one (HDM1) HDM respectively. Amplification of (C) SSR5 and (D) SSR6 with gDNA of common skin pathogens including S. pyogenes, S. aureus, P. acnes, T. rubrum, T. interdigitale and Trichophyton sp. Negative control = water; Positive control = neat mite gDNA (n = 5). (PDF) [file pntd.0009149.s002.pdf]

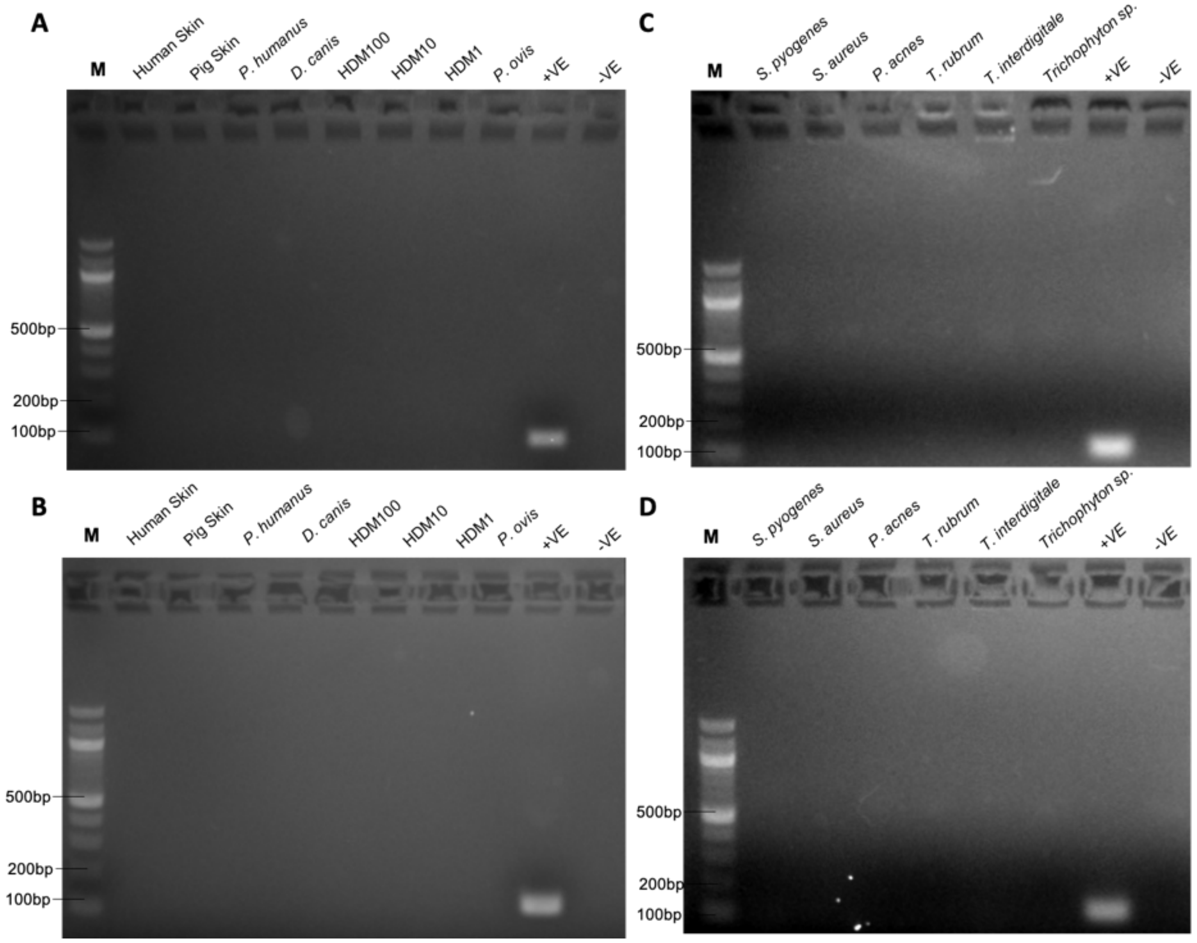

S2 Fig. SSR5 and SSR6 Primer-Specificity. Amplification **(A)** SSR5 and **(B)** SSR6 with gDNA panel consisting of human skin, pig skin, *P. humanus, D. canis, P. ovis* and serially-diluted HDMs. Neat, 1:10, 1:100 dilutions replicate presence of 100 (HDM100), ten (HDM10) and one (HDM1) HDM respectively. Amplification of **(C)** SSR5 and **(D)** SSR6 with gDNA of common skin pathogens including *S. pyogenes, S. aureus, P. acnes, T. rubrum, T. interdigitale* and *Trichophyton sp.* Negative control = water; Positive control = neat mite gDNA (n = 5).
