# Supplementary material for: Molecular diagnosis of scabies using a novel probe-based polymerase chain reaction assay targeting high-copy number repetitive sequences in the Sarcoptes scabiei genome
Source: PLoS Negl Trop Dis. 2021 Feb 24;15(2):e0009149. doi: 10.1371/journal.pntd.0009149 (PMC7939366; doi:10.1371/journal.pntd.0009149)
Supplement: S1 Table — (PDF) [file pntd.0009149.s003.pdf]

S1 Table. Summary of the 2018 IACS criteria for the diagnosis of scabies

**A: Confirmed Scabies**
At least one of:<br>1. Mites, eggs or faeces on light microscopy of skin samples<br>2. Mites, eggs or faeces on visualized on individual using high-powered imaging device<br>3. Mite visualized on individual using dermoscopy
**B: Clinical Scabies**
At least one of:<br>1. Scabies burrows<br>2. Typical lesions affecting male genitalia<br>3. Typical lesions in a typical distribution and two history features
**C: Suspected Scabies**
One of:<br>1. Typical lesions in a typical distribution and one history feature<br>**2.** Atypical lesions or atypical distribution and two history features
**History features**
1. H1: Itch<br>**2.** H2: Close contact with an individual who has itch or typical lesions in a typical distribution

Notes:

1. These criteria should be used in conjunction with the full explanatory notes and definitions (in preparation).
2. Diagnosis can be made at one of the three levels (A, B and C).
3. A diagnosis of Clinical and Suspected scabies should only be made if other differential diagnoses are considered less likely than scabies.
