# Supplementary material for: Molecular diagnosis of scabies using a novel probe-based polymerase chain reaction assay targeting high-copy number repetitive sequences in the Sarcoptes scabiei genome
Source: PLoS Negl Trop Dis. 2021 Feb 24;15(2):e0009149. doi: 10.1371/journal.pntd.0009149 (PMC7939366; doi:10.1371/journal.pntd.0009149)
Supplement: S2 Table — (PDF) [file pntd.0009149.s004.pdf]

S2 Table. Primer and probe details for qPCR assays

Name	Function	Target	Sequence (5' - 3')	Amplicon Size (bp)
WDeg_Cox1F	Forward primer	*cox1*	TGCTATRATTTCTATTGCAACYTTAGG	121
WDeg_Cox1F	Reverse primer	*cox1*	GGRACAGCRATAATTATAGTAGCTGAA	
WDeg_Cox1P	TaqMan probe	*cox1*	FAM-TTTACTGTT/ZEN/GGATTAGATGTTGA TACT/3IABkFQ	
SSR5_F	Forward Primer	SSR5	GTCGAAGGTGGAGACAAAGATAG	118
SSR5_R	Reverse Primer	SSR5	CGGACTCTTCATCGATTGGTATC	
SSR5_P	TaqMan Probe	SSR5	56FAM-TCGTTGAGA/ZEN/TGAAGCGACCAGGA GATG/3IABKFQ	
SSR6_F	Forward Primer	SSR6	TTGCTAGACCCTGATACCAAAC	129
SSR6_R	Reverse Primer	SSR6	ACCAGGAGTTGTTTGACACAATA	
SSR6_P	TaqMan Probe	SSR6	56FAM-AATGCTCTT/ZEN/GCTAAGAGTGATGAT/3IABKFQ	

6-FAM is 6-carboxyfluorescein fluorescent dye; 3IABkFQ is Iowa black quencher
