# Supplementary material for: Molecular diagnosis of scabies using a novel probe-based polymerase chain reaction assay targeting high-copy number repetitive sequences in the Sarcoptes scabiei genome
Source: PLoS Negl Trop Dis. 2021 Feb 24;15(2):e0009149. doi: 10.1371/journal.pntd.0009149 (PMC7939366; doi:10.1371/journal.pntd.0009149)
Supplement: S3 Table — (PDF) [file pntd.0009149.s005.pdf]

S3 Table. RepeatExplorer2 identified repetitive DNA elements with at least one high confidence call amongst four Illumina generated mite sequences

Cluster	Consensus Sequence	Probability Confidence Score (Genome Proportion Score)			
		Human 1	Human 2	Pig 1	Pig 2
606bp LTR (SSR6)	ATTGGTGACTATGAGAAGCCTAGAAA<br>GACTGGTGAAACAATAACAAAAATG<br>AGAGATCCAACTGGTAAAACTGTGAC<br>AAGGATTGTGAAACGAAAACCTGATG<br>GCACTGAAACTGTTGAAGAGCATATT<br>GAAGAACGTAAATCCGTTCCAAGATC<br>ATTGAGCAGAGACGATAAACCTGAGC<br>AGTCGAAAATATTCGATGCCAAAAAA<br>CCGAGAAAGATCGTCAGAATCATTCT<br>GAAGAAACCCAATGGTGATGAATCA<br>GTTGAAGAGCTATTGCTAGACCCTGA<br>TACCAAAC[1]AGGAAGATATCGATAAA<br>GTGTTGAAAGACGAGATACAGAATGC<br>TCTTGCTAAGAGTGATGAT[2]CCGGATG<br>CTAAGATAATTGCCAATATTGTGTCA<br>ACAACTCCTGGT[3]GTAGAAGAGACGG<br>TTGAAGAATTCGAGACGGATCCTCAA<br>ACTGTGAAAAAGAATATTGATGATGT<br>ACTGAAAAAAGCTCCAAGGACCAAA<br>ACCTCGAGAAAGAAAGATCCTCTCGG<br>TAAAATTGTTACTAAGGTATTTAAAC<br>GCAAACCTGATGGCAAAGAAACAGTT<br>GAAGAGAAAGTCGAAGATCCTCAAG<br>TGACCGGGAAAG	0.889 (0.018%)	0.976 (0.021%)	0.959 (0.027%)	0.933 (0.023%)
201bp Microsatellite	TCGTAACTGATATCGAAACTGGCAAA<br>AAGAAAATTGTGAAGAAGAAGAAAC<br>CGAAACAAGGTGTAGATGCAGAAAC<br>ACCTATTGAACAAGAAACAAAAGCA<br>AAAGATGAATCTAAACCAGATGAGTC<br>TGTGCCTGAGATCAGTGAGGTGGTTC<br>CTGAATCACTCGACAAAGATGAAACT<br>AGAGAGGAATTCATCACTGAAG	0.986 (0.057%)	0.994 (0.073%)	0.979 (0.086%)	0.994 (0.086%)
93bp Microsatellite	TACACTTGCTCCTGTTGGTGCAACTG<br>GTCCAACTGAGAAAGAACCATTCACA<br>ACTGAATATCCATCGACTTCGACAAT<br>CTCAGACAAAGATAGAACCACTTATG<br>AACCTGGAACCAG	0.994 (0.026%)	0.993 (0.032%)	0.979 (0.039%)	0.965 (0.037%)

375bp Microsatellite (SSR5)	GTTGGTTTCTCCATTCCACCATCGATT<br>GGTTTTTCGGACTCTTCATCGATTGGT<br>ATC[1]TTGCGATCTTCATCTCCTGGTCG<br>CTTCATCTCAACGA[2]TTTCCTCACTAA<br>TCTCAGTAGTCACAGATTCAGTTCTAT<br>CTTTGTCTCCACCTTCGAC[3]CAGTGTT<br>GGTTTCGAATCTTTGCGTTTGGTCAAT<br>TTTACTTTTCTCTTAGTTTCAATTTCG<br>ACTGTTGATTCTGTTTTAGGAACTTCT<br>TCTTGTTCTTCTGGAGCGATAGATTCT<br>CTTCGCTTGAGATCTATAGAGTCTTCA<br>GTGATTTGCTTTTCTTCTTCAACTGGT<br>TTCTTATGAGAATCTTTTCGTTTGATT<br>TTTCTAGGTTTCTTCGTTTCTTCATCA	0.767 (0.024%)	0.853 (0.034%)	N/A	0.928 (0.044%)
117bp Microsatellite	TACACTTGCTCCTGTTGGTGCAACTG<br>GTCCAACTGAGAAAGAACCATTCACA<br>ACTGAATATCCATCGACTTCGACAAT<br>CTCAGACAAAGATAGAACCACTTATG<br>AACCTGGAACCAG	0.986 (0.057%)	0.981 (0.018%)	0.994 (0.023%)	0.986 (0.030%)
333bp Microsatellite	TTTCGCCTTCCACTTTGGGAATATCAA<br>CATCAACATCTACTTCAGGACCAGAC<br>ACTTTTGGTCCTTTGAACCCAAATTTA<br>GGCATAGAGAACTCCCAATCTTTTCC<br>ATCTTTCTCTTTCGATTTTTTGTCCTTC<br>TTCTTCTTCAGAGATAGATTCACATCA<br>CCTTCAACCTTCGGTAATTCTACATCT<br>GGCATATCAACTTCAGCCTTTCCTAA<br>ATCTACATCTGGTCCGCTTATTTTCGG<br>ACCTTTGAAACCGAATTTGGGCATAT<br>GTAATCCCCATCCTTTGCCATCTTTGT<br>CTCCCATATCTGGTGATTTTCTATCTA<br>AACTCAGATCGA	0.955 (0.013%)	0.981 (0.013%)	0.959 (0.018%)	0.985 (0.018%)
40bp Microsatellite	GGCAAAGAAGCTCCAACCAAGCCTG<br>ATGACAAGAAAGAACCA	0.487 (0.026%)	0.781 (0.012%)	0.056 (0.094%)	0.908 (0.026%)
315bp Microsatellite	TTACAAGAATTGCAAGATGTTGTAGT<br>GTCAAAAGGTCAACCTGTAGCAAAAC<br>TTGAAGCACAAATTGCTGGAAATCCG<br>AAACCAAAGGTCGAATGGTTCAAAG<br>ATAATCAAAAACTTGAAGAATCGCCA<br>CATTTGAAGATGGTCAGCGATGGCAA<br>AGACAAATACTCATTGACTATTCAAA<br>ACGTGAATAGTGAAGACGTTGGACAA<br>TACAAAATTCTGGCCACCAATGATCT<br>TGGTAAAACTGAATCAAAGGCCAAAT<br>TATCAATCGGTGATGGATCAAAACCA<br>GATGATGCAAAGAAACACTCTCCTGA<br>AATT	N/A	N/A	0.959 (0.010%)	0.976 (0.014%)

75bp Microsatellite	GATGGTTGCAATGATGATGTCGGAACACCAGCCAAAGCGCTAGGATCTCTTGCTGATGGTTCTCTGCCTAATTCT	N/A	N/A	0.644 (0.013%)	0.915 (0.014%)
336bp Microsatellite	GGTGGGTCCCTCCCTAGCTGGCGAAGGGTGGGGTGGTGGGGCCAGTGCACACAAGGCCTTTTCAGGCCCCATCCGCCTTCTCCCTGAAGTAGAGCACGTTTGGAGTTGGTTTCCAGCTCCAGCGACGACCTGCAAAGCACCAGTGCACAGGGAGCAGGAGGCAGCCCAGAACCTCCTTGTTCCTATGGCGAGCAATGGGCTAGGGGAGAAACCAGAAAGCGCTGCTTTCCTGAGCAAACACGCATTGGGGCTGAGCTTGGTTTCCCGAGGAGAGTTTCACTTTCGCGAATGCAGCTCCCAGAAGCTAGTTTTGTGCCTTTTCTGAGATGGGCCCTA	N/A	N/A	0.868 (0.050%)	0.071 (0.026%)
84bp Microsatellite	GAGCTTCTTTGCCTGGTTCTTTCTTGTCTTCAGGCTTGGTTGGAGCATCTTTGCCTGGTTCTTTCTTGTCATCAGGCTTGGTTG	N/A	N/A	0.819 (0.024%)	N/A

N/A - not available

[1]Forward primer binding site
[2]Probe binding site
[3]Reverse primer binding site
