# Supplementary material for: Molecular diagnosis of scabies using a novel probe-based polymerase chain reaction assay targeting high-copy number repetitive sequences in the Sarcoptes scabiei genome
Source: PLoS Negl Trop Dis. 2021 Feb 24;15(2):e0009149. doi: 10.1371/journal.pntd.0009149 (PMC7939366; doi:10.1371/journal.pntd.0009149)
Supplement: S4 Table — (PDF) [file pntd.0009149.s006.pdf]

S4 Table. qPCR results for the two-fold dilution series used to determine the assays limit of detection*

Target	Dilution (copies/µL)	Cycle quantification (Cq) values						% Positive
**SSR5**	30	31.39	32.33	31.73	31.99	31.84	32.01	**100**
	15	32.88	32.60	32.85	32.86	33.6	32.83	**100**
	7.5	33.13	33.98	33.62	33.89	35.51	33.15	**100**
	3.75	34.42	35.76	36.65	34.11	35.89	35.74	**100**
	1.875	N/A	36.23	35.96	N/A	N/A	N/A	**33.3**
	0.9375	36	37.75	36.74	34.68	N/A	N/A	**66.7**
**SSR6**	30	31.25	30.80	30.93	30.82	30.72	31.35	**100**
	15	32.16	31.47	32.26	31.8	31.44	32.04	**100**
	7.5	34.41	32.68	34.21	36.19	32.47	33.56	**100**
	3.75	32.77	33.54	34.12	34.73	34.04	35.73	**100**
	1.875	35.89	35.6	36.67	36.67	36.16	34.15	**100**
	0.9375	36.48	N/A	36.84	N/A	N/A	37.33	**50**
cox1	30	31.73	31.65	31.71	32.11	31.85	31.74	**100**
	15	33.42	33.01	32.81	33.12	33.67	33.34	**100**
	7.5	34.54	35.81	33.64	33.59	33.24	33.4	**100**
	3.75	36.09	34.58	35.94	33.71	33.92	35.86	**100**
	1.875	N/A	N/A	37.89	36.3	N/A	NA	**33.3**
	0.9375	N/A	35.97	38.51	37.87	36.37	N/A	**66.7**

**Two dilution series were prepared, and triplicates of each dilution were analysed with qPCR.*

*** Positive is determined by any reaction reporting a Cq value.*

N/A - not available
