# Supplementary material for: Molecular diagnosis of scabies using a novel probe-based polymerase chain reaction assay targeting high-copy number repetitive sequences in the Sarcoptes scabiei genome
Source: PLoS Negl Trop Dis. 2021 Feb 24;15(2):e0009149. doi: 10.1371/journal.pntd.0009149 (PMC7939366; doi:10.1371/journal.pntd.0009149)
Supplement: S5 Table — (PDF) [file pntd.0009149.s007.pdf]

S5 Table. Triplicate cycle quantification (Cq) values for mite extractions to determine assay sensitivity

**Cq – cycle quantification*

	**Cq values of the developed qPCR assays***						**Cq values of the reference qPCR assay***		
**No. of mites per reaction**	**SSR5**			**SSR6**			***cox1***		
1	24.04	24.01	24.29	26.43	26.69	26.47	24.63	24.78	24.65
0.1 (dilution 1:10)	27.99	28.05	27.75	29.42	29.72	29.77	28.75	28.92	28.8
0.01 (dilution 1:100)	30.26	32.29	32.08	32.53	32.51	32.23	32.49	31.55	32.21
0.001 (dilution 1:1000)	N/A	N/A	N/A	N/A	N/A	N/A	N/A	N/A	N/A

***SSR5, SSR6 and cox1 are the genes targeted in the assays*

N/A - not available
