# Supplementary material for: Molecular diagnosis of scabies using a novel probe-based polymerase chain reaction assay targeting high-copy number repetitive sequences in the Sarcoptes scabiei genome
Source: PLoS Negl Trop Dis. 2021 Feb 24;15(2):e0009149. doi: 10.1371/journal.pntd.0009149 (PMC7939366; doi:10.1371/journal.pntd.0009149)
Supplement: S6 Table — (PDF) [file pntd.0009149.s008.pdf]

S6 Table. Profile of school children from StoP Trial in Kimberley, WA enrolled in this study.

Individual No.	Age	Indigenous?	Patient Diagnosis	Sample Type	Treatment	Sample Source
1	13	Yes	Suspected scabies	FLOQ swab	No	Kimberley, WA
2	10	Yes	Suspected scabies	FLOQ swab	No	Kimberley, WA
3	10	Yes	Suspected scabies	FLOQ swab	No	Kimberley, WA
4	8	Yes	Suspected scabies	FLOQ swab	No	Kimberley, WA
5	10	Yes	Suspected scabies	FLOQ swab	No	Kimberley, WA
6	6	Yes	Suspected scabies	FLOQ swab	No	Kimberley, WA
7	7	Yes	Suspected scabies	FLOQ swab	No	Kimberley, WA
8	9	Yes	Suspected scabies	FLOQ swab	No	Kimberley, WA
9	7	Yes	Suspected scabies	FLOQ swab	No	Kimberley, WA
10	5	Yes	Suspected scabies	FLOQ swab	No	Kimberley, WA
11	5	Yes	Suspected scabies	FLOQ swab	No	Kimberley, WA
12	8	Yes	Suspected scabies	FLOQ swab	No	Kimberley, WA
13	7	Yes	Suspected scabies	FLOQ swab	No	Kimberley, WA
14	5	Yes	Suspected scabies	FLOQ swab	No	Kimberley, WA
15	6	Yes	Suspected scabies	FLOQ swab	No	Kimberley, WA
16	9	Yes	Suspected scabies	FLOQ swab	No	Kimberley, WA
