# Supplementary material for: Molecular diagnosis of scabies using a novel probe-based polymerase chain reaction assay targeting high-copy number repetitive sequences in the Sarcoptes scabiei genome
Source: PLoS Negl Trop Dis. 2021 Feb 24;15(2):e0009149. doi: 10.1371/journal.pntd.0009149 (PMC7939366; doi:10.1371/journal.pntd.0009149)
Supplement: S7 Table — (PDF) [file pntd.0009149.s009.pdf]

S7 Table. Diagnostic results of StoP Trial samples by qPCR

	Suspected scabies (n = 15)	No scabies (n = 0)
**Clinical Assessment**		
	15	0
**qPCR (SSR5)**		
	1	14
**qPCR (*cox1*)**	2	13
