# Supplementary material for: Molecular diagnosis of scabies using a novel probe-based polymerase chain reaction assay targeting high-copy number repetitive sequences in the Sarcoptes scabiei genome
Source: PLoS Negl Trop Dis. 2021 Feb 24;15(2):e0009149. doi: 10.1371/journal.pntd.0009149 (PMC7939366; doi:10.1371/journal.pntd.0009149)
Supplement: Attachment — Submitted filename: Responses to ReviewersComments_291020.docx [file pntd.0009149.s010.docx]

Reviewer #1:

1. The results of this study show an improvement in diagnostic laboratory detection of scabies using PCR over standard microscopic examination of skin scrapings. The work was thorough and presented clearly.  However, after reading the paper, I was left wondering what the role of laboratory diagnosis is in this setting since even with very sensitive molecular techniques, there was a gap between positive lab tests and clinical suspicion based on standardized criteria. If there is clinical suspicion but a laboratory test, even a highly-sensitive molecular test, is negative, how would a clinician respond?
   a. *Confirmation of diagnosis would still depend on a combination of standardised clinical diagnostic criteria, and molecular testing. Training for clinical observations and quality sample collection for molecular testing would also need to be provided. We agree that further validation studies are required (Please see Discussion, p36; lines 563-573).*
2. Although an improvement, PCR may still not be a totally reliable laboratory test. I think that the background information needs to more clearly state what the role of laboratory testing is given the low sensitivity even with the new molecular test described.
   a. *We agree that the role of a laboratory test such as PCR should be clearly stated. However, further validation would still be required to be carried out for a clearer role to be established. We have aimed to provide this laboratory test as a complement test to clinical diagnosis, and have stated its potential role in the final paragraph of the Discussion (pages 33-34).*
3. p.9 “Carer” to “Caregiver”.
   a. *“Carer” changed to “Caregiver” in Methods (Ethics statement): lines 142 and 145.*
4. Define NA in Tables S3, S4 and S5.
   a. *NA defined as “not available” in Supplementary Information; new Tables S4, S5 and S6 (Table legend)*
5. p.22 Fig 4 Legend:”…before sample were taken…” to “… before samples were taken…”.  The same correction is needed in the legend for Table 2.
   a. *Addressed in Fig. 4 legend and Table 2 legend.*
6. Legend for Table 2, “ifestation” needs to be corrected to “infestation”.
   a. *Addressed in Table 2 legend.*

Reviewer #2:

1. This study reports on the use of a new PCR method and the use of a new type of swab (as an alternative to skin scrapings) for the diagnosis of S. scabiei infections. This is a good study and by a well renowned group working in the difficult area of scabies diagnosis and treatment. Diagnosis of scabies/mange is a persistent challenge for humans and many animal species, and advancements in diagnosis are therefore of significant value. It is notable that improvements in the successful collection of mite material from infected individuals is perhaps the most valuable advancement that can be made for S. scabiei diagnosis. While this study focusses primarily on diagnostic method improvement, it also contributes some advancement in improved collection of mite material.

General feedback

2. To me the demonstrated value of FLOQ swabs is arguably the most important contribution of this study, and could perhaps be emphasised more strongly (see above and my specific comments).
   a. *We have added a statement in the first paragraph of the Discussion (page 30; Lines 518-520) to emphasise the value of FLOQ swabs demonstrated in this study.*
3. The biggest challenges I encountered reading this manuscript was keeping track of the varied components/sections of the study, and also variation in reporting results across the components, which wasn’t always clear as to why. Some of my specific feedback below reflects these challenges.
   a. *For clarity, we have included an overview of our research project represented as a flow diagram (S1 Figure). Details stated in point 6.*
4. Given the main limitation to diagnosing S. scabiei infection in patients is the acquisition of mite material (through skin scrapes or swabs), it would seem intuitive to always bring results back to the amount of mite material needed for a positive diagnosis. By this I mean represent all Cq and DNA copies results also as the amount of mite material required for diagnosis. You do this in Figure 2, but it should also be in Figure 3, Table 5 and through the results and discussion text (most notably p21, 27, 31).
a. *At this stage, we are unable to translate Cq values and DNA copies to amount of mite material needed for a positive diagnosis. A large-scale validation study would be required to accurately compare mite numbers to Cq values. With the limited testing of our optimized qPCR assays in clinical and field samples, any comparisons made at this stage may be invalid. However, we were able to show in our study that our qPCR assays are highly sensitive, that is, we successfully amplified DNA from 10-fold dilutions of a single mite (Please see Fig 2). This presents evidence of our qPCR assays’ potential to detect minimal mite material.*
5. The section of this study on Aboriginal school children is quite inconclusive and therefore a limitation of the study. It read to me that either: sample collection was insufficient for a fair PCR diagnosis; or more samples per individual are needed for a higher detection probability; or the criteria for a clinical diagnosis was not applied as well as needed. I do not know the answer to this, and I was left wondering how meaningful this section was. If it is so inconclusive, should it even be included in the study?
   a. *Data from the Kimberley dataset was unfortunately inconclusive. However, this study underlies the importance and challenges faced when conducting clinical validation studies of a sufficient scale, and supports the need for an appropriately designed validation study. We have now moved the results of the Kimberley study in the Supporting Information Section (S9 Table).*

Specific feedback

6. There are quite a few components to this study, types of samples collected, and testing undertaken. I think the ms could be made clear through the inclusion of a workflow diagram documenting these details.
   a. *We have now included a workflow diagram to present an overview of our project in the Supporting Information Section (S1 Figure).*
7. Methods: I note repetition of content in the Ethics Statement and Study Design sections. These paragraphs need revision to eliminate the repetition.
   a. *­­­We have now revised the Ethics Statement (page 9; paragraph 2) to avoid the repetition of content in Study Design, noted by the reviewer.*
8. Table 2: Change the “Diagnosis” column heading to “Clinical diagnosis”.
   a. *Addressed in Results: Table 2.*
9. P25. It is surprising that you found qPCR on FLOQ swabs to be more sensitive for detecting S. scabiei than qPCR on skin scrapings. This would seem contrary to previous literature, and I was left wondering if this result was genuine or more a reflection of the way the sentence presented the results. Assuming it is genuine, this is an important diagnostic method result. It would be valuable to present a table comparing positives and negatives between the methods, and associated sensitivity and specificity.
   a. *We have presented qPCR results on clinical samples collected by FLOQ swabs in Table 3 with associated sensitivity and specificity. The qPCR results on clinical samples collected by skin scrapings on the other hand, are presented in Table 4. In addition, Table 5 also shows qPCR performance being more sensitive in detecting S. scabiei on samples collected by FLOQ swabs than skin scrapings when we compared our assays’ performance on clinically diagnosed scabies cases. We therefore believe that collectively these results show that this is a promising diagnostic method.*
10. Table 3: Why not report the pig model results in this table also? Afterall, you have 24 samples (spanning four individuals) from pigs, plus controls.
    a. *For assay development, we tested the efficacy of FLOQ swabs as a sample collection method on the pig models. In Table 3, we present the qPCR results of testing the swabs on clinical human samples hence, we did not combine the results.*
11. Discussion, paragraph 1: It is neither surprising nor novel that PCR was more sensitive than microscopy. For this reason, I think a real value of this study is in reporting the use of FLOQ swabs.
    a. *We have now highlighted our developed qPCR assays more sensitive performance on clinical samples collected by FLOQ swabs compared to those collected by skin scrapings on the first paragraph of the Discussion (Lines 518-520).*
12. Discussion, paragraph 3: Was qPCR done on skin scrapings to compare with swabs? This is a more meaningful comparison than microscopy for methods improvement. See also my comments regarding p25.
    a. *qPCR was performed on samples collected both by swabs and skin scrapings. Please see results presented in Tables 3 and 4 with associated sensitivity and specificity. Data presented in Table 5 further supports this comparison. (qPCR assay performance was more sensitive in detecting S. scabiei on samples collected by FLOQ swabs than skin scrapings from seven clinically diagnosed scabies cases).*
13. Discussion, paragraph 4: See my above comments about this being a major limitation of the study. (Kimberley data)
    a. *We do agree on the limiting dataset from Kimberley in this study. However, we wish to highlight the difficulty of conducting rigorous studies in field settings such as the SToP trial. Given that our co-investigators gave us the opportunity to conduct this work to test our FLOQ swabs in a remote community setting we felt that it was important to publish the data. As we note above, the results further highlight the importance of conducting larger scale validation studies of our qPCR assays and FLOQ swabs to investigate and potentially mitigate its limiting factors.*
14. Discussion, final paragraph: The authors should also highlight that multiple swabs may need to be taken per individual to improve the detection probability for S. scabiei. This appears to be an underrepresented consideration (and valuable future direction) for use of FLOQ in S. scabiei diagnosis.
    a. *We have now revised the final paragraph of the Discussion (pages 33-34; lines 600-611) to highlight the need for multiple swabs per individual to improve the detection probability of S. scabiei in future validation studies planned.*
15. The authors might also note LAMP as an additional diagnostic method evaluated for S. scabiei: Fraser et al. PeerJ 6:e5291.
    a. *Citation for LAMP has been included in PCR-based scabies diagnostic assays in paragraph 2 of Introduction.*

Reviewer #3:

1. The authors present promising findings but correctly indicate that a larger validation study needs to be performed to better estimate diagnostic sensitivity. There are no apparent ethical concerns.
2. The results are clearly expressed. Image quality for Figure 1 and 3 needs improving.
   a. *We have now improved image quality of Fig 1 and 3.*
3. The conclusions are supported by the data and limitations of the study are well discussed. The public health relevance is highlighted well.
4. This paper presents novel preliminary findings of a newly developed qPCR assay, combined with a non-invasive sampling technique, to enhance the diagnosis of scabies. The public health importance of such research in scabies is very high. The authors acknowledge the limitations of the present study and plan a larger validation study.

Reviewer #4:

1. This is an interesting paper outlining the development and assessment of 2 PCR assays for the diagnosis of scabies. The authors also highlight the superior performance of FLOQ swab samples when using PCR assays for scabies diagnosis.
2. ABSTRACT - Include sensitivity in other clinical settings (treated patients/school children)
   a. *We are unaware of large scale studies discussing diagnostic standards amongst treated scabies patients and school children involving swab sampling/skin scrapings with molecular assays.*

Author summary -requires revision

3. "for progammatic use" - consider excluding.
   a. *Removed from line 64.*
4. "Through new insights into.." - revise sentence - "We developed two PCR based diagnostic assays targeting..... based on new insignts..."
   a. *Addressed in lines 67- 68.*
5. "The use of such elements" - reword for clarity - maybe split into two sentences?
   a. *Addressed in lines 68- 69.*
6. deleted "established"
   a. *Addressed in line 70.*
7. revise "should provide a definitiive diagnosis in a clinical context" - perhaps "may provide a useful additional tool in the clinical diagnosis of scabies".
   a. *Addressed in lines 72-73.*

Introduction

8. re-word "diagnostic deficiencies" - is this in reference to "the limitations of available methods of diagnosis"
   a. *Addressed in line 77.*
9. define abbreviation "qPCR"
   a. *Addressed in line 99.*
10. review "However, only the assay targeting the mitochondrial..." - perhaps "To date, only the assay...".
    a. *Addressed in line 103.*

Methods

11. clarify who performed microscopy on skin scraping samples.
    a. *Addressed in lines 292-293.*

Results

12. re "Comparative testing of FLOQ and Catch all swabs" - presumably the values for DNA copies are mean values? (7351.1 and 3782,1 etc) - clarify in text.
    a. *Yes , DNA copies reported are mean values of triplicate reactions and now stated on page 21; lines 401-402.*
13. please present overall sensitivity in Darwin context (treated and non-treated combined)
    a. *Sensitivity of treated and non-treated patients cannot be combined as overall sensitivity of patients in different conditions will not be an accurate representation of infection status. Treated patients can retain mite material even after treatment, which can lead to false positives. Classifying them would thus ensure a lesser risk of that. More studies will have to be conducted to evaluate the detection kinetics.*
14. can you calculate a sensitivity for the Kimberley school children's context? compared with clinical diagnosis - later discuss limitations of gold standard as suggested.
    a. *While formal display of the very low calculated sensitivity is possible, we have concerns about the accuracy of the clinical diagnosis, thus highlighting the need for a formal validation study using gold standard parasitologic tests or dermatoscopy.*
15. the sentence "Lower sensitivity of the qPCR assays..." is incorrect with regard to treated patients (sens 40% for skin scrapings was the same as for swab) - revise sentence.
    a. *We have now corrected this statement. Please see page 25; Lines 469-471).*

Tables

16. table 3- present overall numbers (treated and non-treated combined) - consider including PPV and NPV (as outlined in methods?).
    a. *PPV and NPV were not included and thus, their description removed in the methods section. Overall numbers of treated and non-treated patients cannot be combined (See point 13 above).*
17. table 4 - some overlap with table 3 - consider combining - alternatively exclude columns for "no scabies" - but rather report total - consider providing percentage positive in brackets next to numbers for microscopy and qPCR
    a. *For clarity, we have now removed Table 4 and replaced with a new Table 4 showing Scabies diagnosis results by qPCR on samples collected by skin scrapings to compare qPCR results on samples collected by FLOQ swabs presented in Table 3. qPCR assay detection and positivity rate are reported as assay sensitivity in both Tables (3 and 4). The sensitivity of scabies diagnosis by microscopy is described in the text (page 25; lines 471-473).*
18. table 6 - doesn't add anything over and above text - consider excluding
    a. *Table 6 has been moved to SI Table 8, as a supplementary table for reference.*
19. tables 7 - again doesn't add anything over and above text  - I would exclude
    a. *Table 7 has been moved to SI Table 9, as a supplementary table for reference*

Discussion

20. re the sentence "This was ovserved in clinical patients from Darwin who were diagnosed with an early infection of scabies" - was this included in the results - if not avoid introducing new data in the discussion.
    a. *We have deleted this statement and revised the Discussion in paragraph 4.*
21. discuss with more clarity the possibility of "misclassification" of scabies diagnosis in the context of the schools study - perhaps with a reference on the limitations of clinical diagnosis?
    a. *We have now revised paragraph 4 of the Discussion to clarify this point (pages 31-32; lines 548-561)*
22. revise the sentence "A confirmatory test would be very useful to evaluate the success of scabies treatment" - given the relatively low sensitivity and the possibility of persistent DNA in treated cases I'm not sure the results support this statement
    a. *We have revised paragraph 6 of Discussion (pages 32-33; lines 575-585) to address the reviewer’s comment.*
23. include more complete discussion of the limitations of this study including: small numbers, heterogenous population assessed (treated / non-treated), lack / limitations of "gold standard" diagnostic method / reliance on clinical diagnosis, etc
    a. *We have revised the Discussion accordingly, as suggested by the reviewer in the context of the limitations of our study described above.*
24. in concluding remarks (final paragraph) - and in the discussion generally - re-iterate the implications of the findings of this study in the context of scabies diagnosis - my take from reading is that this may have an additional role in improving accuracy of clinical diagnosis of scabies? possibly have a role in follow up of treatment? - not sure of role in large scale diagnostic studies but this warrants further investigation.
    a. *Revisions have been made to the final paragraph of the Discussion (pages 33-34) to address the reviewer’s comments*
