# Supplementary material for: Molecular diagnosis of scabies using a novel probe-based polymerase chain reaction assay targeting high-copy number repetitive sequences in the Sarcoptes scabiei genome
Source: PLoS Negl Trop Dis. 2021 Feb 24;15(2):e0009149. doi: 10.1371/journal.pntd.0009149 (PMC7939366; doi:10.1371/journal.pntd.0009149)
Supplement: Attachment — Submitted filename: Scabies Rejoinder2.docx [file pntd.0009149.s011.docx]

5 January 2021

Richard Stewart Bradbury, PhD

Associate Editor

PLOS Neglected Tropical Diseases

Michael Marks

Deputy Editor

PLOS Neglected Tropical Diseases

Dear Dr. Bradbury and Dr. Marks,

Thank you for giving us the opportunity to revise our manuscript “ Molecular diagnosis of scabies using a novel, probe-based polymerase chain reaction assay targeting high-copy number repetitive sequences in the *Sarcoptes scabiei* genome”, based on further comments and requests raised by Reviewer # 2 on our initial resubmission.

We are now re-submitting a significantly revised version of our manuscript addressing Reviewer #2’s comments.

Please find below our responses and a description of the changes we have made in the manuscript *(in italics)*:

Reviewer #2: Thank you for the opportunity to review this manuscript. I think that it has potential to make valuable contributions to (1) the manner in which S. scabiei are sampled from patients and (2) the subsequent diagnostic testing of the samples.

I thank the authors for the manuscript revisions they have made. This has improved the study. I am a little disappointed that the authors showed resistance some of my recommended revisions. I was left with the impression that some justifications for not considering appeared more as excuse making, and some responses where changes were made were a bit minimal. Below are the recommendations I made on the original review (or follow-on comments) that I would like the authors to consider more carefully.

To me the demonstrated value of FLOQ swabs is arguably the most clinically impactful contribution of this study, and could be emphasised more strongly.

*We agree with the reviewer and have made changes to increase the prominence of this finding (Please see Abstract: Lines 53-56; Results:* *Lines* *323-479: we have made significant changes in this section to present clearly our significant findings: the use of FLOQ swabs being superior to skin scrapings for a positive qPCR diagnosis applying our new target sequences; Discussion ). However, we consider that these findings are preliminary and will require further and larger study.*

In my original review I recommended the authors consider using the relationship between Cq values and amount of mite material presented in figure 2 to convert other Cq values (fig 3, table 5, p21, 27 and 31) to amount of mite material. My reasoning is that this is ultimately the most practical piece of information to someone attempting to sample mites. The authors have responded with a justification that this is not possible without additional research. I am still not entirely clear on that justification, since Cq values they report throughout the manuscript are consistent with the range presented in figure 2 and ‘estimated amount of mite material’ would seem a reasonable metric to report. I would like the authors to consider this more closely. If they really feel strongly that they cannot do this, then at the very least a paragraph in the discussion is needed about it.

*We agree that there is a strong log-linear relationship between concentration of the PCR target and the Cq value (S4 Figure for plasmid target and Figure 2 / S6 Table for serially diluted mite target). We also agree that Cq value can act as a semi-quantitative measure of level of target in any clinical sample (eg data on FLOQ swab vs scraping; Table 3). However, there are a number of important considerations in terms of interpolating the derived amount of mite DNA in a specific sample from the Cq value. Sampling of skin cannot be undertaken in a similar way to quantitative sampling of other biologic matrices (eg: malaria parasites or HIV virus in blood; CFU of bacteria in urine etc). Reporting of the amount of mite DNA in a specific sample will inevitably be subject to sampling bias. Comparison of the quantity of mite material in a swab taken from the ear of a pig versus that from the skin of a patient may not have significant value to report. We believe that future studies, for example documenting the change in assay results after treatment and detailed sampling protocols combined with dermatoscopy may result in improved understanding of the quantitative information present in reporting density of the assay target in the matrix being sampled.*

I am pleased the authors considered my suggestion for a work-flow diagram. They have made a good start on this, and it should be included as a full figure in the manuscript (possibly at the expense of another figure). The format of the figure could be made more condensed to take up less page space, and also more information could be added on sample sizes, reasoning behind steps, key results, etc.

*We thank the reviewer for this recommendation and have reduced the figure size and moved it to the main section. We have also taken the opportunity to re-evaluate all the tables and figures and have made some changes to elevate some and demote others to and from the supplementary. We have deleted tables 3 and 4 and have replaced them with a new Figure (Fig 5a and 5b) describing graphically the study results. We believe that these changes have improved the ability of the reader to interpret the data presented.*

There are a several comments I made that reflect the challenges I had with integrating the results across Tables 3-5, and the authors responses reinforced that for me. Below are those original comments and author responses which illustrate this point. I would like to encourage the authors to re-consider the presentation of these tables to assist the reader with their integration.

*We thank the reviewer. See our description of the changes we have made above.*

1. MY COMMENT: P25. It is surprising that you found qPCR on FLOQ swabs to be more sensitive for detecting S. scabiei than qPCR on skin scrapings. This would seem contrary to previous literature, and I was left wondering if this result was genuine or more a reflection of the way the sentence presented the results. Assuming it is genuine, this is an important diagnostic method result. It would be valuable to present a table comparing positives and negatives between the methods, and associated sensitivity and specificity.

AUTHOR RESPONSE: We have presented qPCR results on clinical samples collected by FLOQ swabs in Table 3 with associated sensitivity and specificity. The qPCR results on clinical samples collected by skin scrapings on the other hand, are presented in Table 4. In addition, Table 5 also shows qPCR performance being more sensitive in detecting S. scabiei on samples collected by FLOQ swabs than skin scrapings when we compared our assays’ performance on clinically diagnosed scabies cases. We therefore believe that collectively these results show that this is a promising diagnostic method.

*Above we have addressed the interesting and unexpected finding of the increased sensitivity of the FLOQ swab, the methodologic difficulties of combining the pig and human sampling, and the changes we have made to the figures and tables.*

2. MY COMMENT: Table 3: Why not report the pig model results in this table also? Afterall, you have 24 samples (spanning four individuals) from pigs, plus controls.

AUTHOR RESPONSE: For assay development, we tested the efficacy of FLOQ swabs as a sample collection method on the pig models. In Table 3, we present the qPCR results of testing the swabs on clinical human samples hence, we did not combine the results.

3. MY COMMENT: Discussion, paragraph 3: Was qPCR done on skin scrapings to compare with swabs? This is a more meaningful comparison than microscopy for methods improvement. See also my comments regarding p25.

AUTHOR RESPONSE: qPCR was performed on samples collected both by swabs and skin scrapings. Please see results presented in Tables 3 and 4 with associated sensitivity and specificity. Data presented in Table 5 further supports this comparison. (qPCR assay performance was more sensitive in detecting S. scabiei on samples collected by FLOQ swabs than skin scrapings from seven clinically diagnosed scabies cases).

*See our comments above.*

We are looking forward to a favourable response.

Sincerely yours,

Cielo Pasay, PhD./ Prof. James McCarthy

Corresponding Authors
